# Quality and Safety Assessment of Edible Seaweeds *Alaria esculenta* and *Saccharina latissima* Cultivated in Scotland

**DOI:** 10.3390/foods10092210

**Published:** 2021-09-17

**Authors:** Anastasia E. Lytou, Eirini Schoina, Yunge Liu, Kati Michalek, Michele S. Stanley, Efstathios Z. Panagou, George-John E. Nychas

**Affiliations:** 1 Laboratory of Microbiology and Biotechnology of Foods, Department of Food Science and Human Nutrition, School of Food and Nutritional Sciences, Agricultural University of Athens, 11855 Athens, Greece; alytou@gmail.com (A.E.L.); irenesxo@gmail.com (E.S.); stathispanagou@aua.gr (E.Z.P.); 2 Department of Food Science and Engineering, Shandong Agricultural University, Tai’an 271018, China; liuyunge@meatsci.com; 3 Scottish Association for Marine Science (SAMS), Oban PA37 1QA, UK; Kati.Michalek@sams.ac.uk (K.M.); Michele.Stanley@sams.ac.uk (M.S.S.)

**Keywords:** macroalgae, microorganisms, spoilage, nutrition facts, drying, rehydration, kelp

## Abstract

Within Europe over the last 10 years, there has been an increase in seaweeds cultivated for human consumption. For food safety reasons, it is important to assess the microbiological and nutritional quality of the biomass. The fresh and dried edible seaweeds *Alaria esculenta* and *Saccharina latissima* were assessed over two consecutive years for the presence of microorganisms. Seaweed samples supplied from Scotland were stored under isothermal conditions for specific time intervals depending on the sample’s condition (fresh, dried or rehydrated). During storage, microbiological analyses were performed for the enumeration of Total Viable Counts (TVC), *Pseudomonas* spp., Enterobacteriaceae and *Bacillus* spp., as well as yeasts and molds. Additionally, bacterial colonies from the Marine Agar growth medium were isolated and subjected to PCR-RAPD analysis for characterization of the bacterial diversity of seaweeds. Bacterial isolates with different fingerprint patterns were further subjected to sequencing (16S rDNA, V1–V4 region). The presence of human pathogenic bacteria was also investigated. Results showed that the initial population of TVC was differentiated depending on the year of seaweed harvest, being closer to the enumeration limit (1.0 log CFU/g) in fresh samples from 2020 and higher in samples from 2019 (6.7 and 3.9 log CFU/g in *A. esculenta* and *S. latissima*, respectively). DNA-based analysis revealed the presence of *Psychrobacter*, *Cobetia* and *Pseudomonas* species in *A. esculenta*, while *Psychrobacter* and *Micrococcus* species were present in *S. latissima*.

## 1. Introduction

In the last 20 years, seaweed production has almost tripled, from 11 million tonnes in 2000 to 33 million tonnes in 2018 [1]. Nevertheless, consumers in Europe are still not very familiar with this type of seafood, despite the existence of old culinary traditions related to seaweed consumption in the coastal communities of many western countries (United Kingdom, Ireland, Iceland, Norway, west coast of France, Canada, etc.) [2,3]. However, the increased interest in healthier diets as well as the preference for more sustainable food sources and production procedures has resulted in an increase in consumers’ interest and further enhancements to seaweed production and marketing around the world [1].

In Asia, there are many popular seaweed species from a gastronomic point of view, belonging to the three major groups of marine macroalgae (Rhodophyta (red), Chlorophyta (green) and Phaeophyceae (brown)), including *Porphyra* (nori), *Ulva* spp. (aonori), *Saccharina japonica* (kombu), *Undaria pinnatifida* (wakame), etc. In Europe, the U.S. and Canada, seaweed farming has particularly focused on species such as *Palmaria palmata*, *Alaria esculenta*, *Saccharina latissima* and *Laminaria* spp., mainly for human consumption as well as for the production of hydrocolloids (alginates, carrageenan, agar-agar) [4,5].

*A. esculenta* (winged kelp) and *S. latissima* (sugar kelp) are two of the most frequently cultivated marine algae species in Europe, characterized by their ability to reach high biomass yield and their abundance in valuable nutritional elements [6,7,8]. *S. latissima*, also known as sweet kombu, contains a substantial amount of mannitol (ca. 14%). Despite its relatively low protein content compared to other seaweed species, it does contain essential amino acids along with several functional bioactive components [9,10]. However, this must be balanced against its high iodine content and the specific strategies needed to address this; as such, this is considered one of the main shortcomings of this species [11]. According to previous studies, *A. esculenta*—amongst its other nutritional benefits—is particularly rich in fucoxanthin, a high-value carotenoid pigment, mainly due to its antioxidant activities as well as its ability to control blood glucose levels [6].

An important step in the development of the edible seaweed industry is the optimization of the procedures during, and particularly after, harvest. Freezing and drying are the two most commonly employed methods for the preservation of edible seaweeds, resulting in a longer shelf life, while the proper handling of the biomass during post-harvest operations should be taken into consideration for the production of high-quality and safe seaweeds [5,10,12].

Seaweed biomass will also contain varying amounts of heavy metals (for example, lead, arsenic, cadmium, mercury), plus other compounds (iodine, pesticide residues, dioxins, antibiotics, drugs, biotoxins, allergens, micro- and nano-plastics). Pathogenic bacteria such as *Salmonella*, *Bacillus*, pathogenic *Escherichia coli*, *Listeria*, *Staphylococcus aureus* or pathogenic *Vibrio* could pose significant hazards related to the safe consumption of seaweeds [13]. However, apart from the safety aspect, little information is available about the microbiological quality and the shelf life of seaweeds. The high nutrient content along with the high moisture level render seaweeds a highly perishable foodstuff [14]. Although previous studies have focused on the microbial associations of several marine algae species [15,16], few studies have investigated the microbiological quality, including the shelf-life and the spoilage potential of edible seaweeds. Among the most characteristic are the quality assessments of *A. esculenta* and *S. latissima* [17], as well as the evaluation of sensory, physicochemical and microbial properties of *Gracilaria* and *Palmaria* [18]. Freshness and shelf-life of *Ulva rigida* were also investigated [19], while several aspects of seaweed quality, including their microbiological profile and shelf-life, were evaluated [14,20].

The aim of the present study was to investigate the microbiological quality of fresh (wet), dried and rehydrated seaweeds belonging to the species *A. esculenta* and *S. latissima*, both after harvest and storage at different temperatures, focusing mainly on the differences between (a) the two different harvest years and (b) the two different seaweed species. Additionally, the seaweed products were examined for the presence of human pathogens while nutritional parameters, such as proteins, carbohydrates, fat, fatty acid profile, moisture and ash content, were determined in order to obtain an overall view on the quality of these two seaweeds.

## 2. Materials and Methods

### 2.1. Seaweed Collection and Treatments

*A. esculenta* and *S. latissima* were cultivated at the Port-a-Bhuiltin seaweed farm operated by the Scottish Association for Marine Science (SAMS, Scotland). Appropriate thalli of both species were hand-harvested using knives to cut the stipe above the blade and placed into clean sampling containers (60 L plastic boxes). An amount of ca. 4 kg per species was transferred into clean plastic bags and stored at −20 °C until shipping. On the day of shipping, the plastic bags were tightly packed into polystyrene boxes and shipped to the Laboratory of Food Microbiology and Biotechnology of the Agricultural University of Athens within 48 h. The samples were transported under refrigeration and the temperature in the packages of all tested batches was below 2 °C. It should be noted that seaweed samples were still frozen on arrival at the laboratory. For the experimental setup, two different batches of seaweed products originating from two different years (2019 and 2020) were tested.

The drying procedure performed at SAMS differed between the two harvest years. In 2019, fresh seaweeds were spread on tarp, allowed to air-dry (exposed to sun and wind), and moved inside overnight and/or in the case of rainy or too windy weather (2–3 days). The final drying procedure was performed inside with heating systems and a dehumidifier (ca. 2 days). Finally, the dried blades were crushed up into small pieces and stored in labelled plastic bags of ca. 0.5 kg each.

In 2020, prior to harvest, a hanging drying grid (set of parallel washing lines) was set up inside the SAMS facilities. On the day of harvest, seaweed blades were laid carefully over the washing lines, making sure that no more than two blades are overlaying each other. The building was constantly heated and ventilated and the seaweed left until completely dry (10–11% moisture—2 days). A clean tarp was placed below the hanging grid and the dried seaweed was stripped off and packed into clean plastic bags for shipping (1 kg per species). The dried samples were subsequently sent to the Laboratory of Food Microbiology and Biotechnology for microbiological and nutritional quality evaluation.

The microbiological load and certain nutritional parameters of the dried samples were evaluated on the day of arrival at the laboratory, as well as after 6 months of storage at 22 °C (microbiological analysis only). The frozen samples were allowed to thaw overnight at 0–1 °C and subsequently divided aseptically into 50 g portions, placed in polystyrene trays and stored aerobically at different temperature conditions. Four samples of each seaweed species, storage temperature and form were analyzed microbiologically at specific time intervals.

The second part of the experimental design included the rehydration of dried samples to simulate a common consumer practice before seaweeds’ consumption. A quantity of dried samples (30–40 g) was soaked in sterile water for 5 min, allowed to drain off, placed in polystyrene trays and stored for 7 days at 5 and 10 °C. Microbiological analyses were conducted at certain time points throughout storage. Detailed information about the experimental design is provided in Table 1 (A and B).

Finally, in parallel with the aforementioned trials, frozen samples of both species (2019 batch) were thawed overnight at 0–1 °C and a sufficient quantity (ca. 100 g) of each seaweed sample was dried in a lab-scale dryer at 40, 50 and 60 °C for 16 h. The experiment was repeated twice while duplicate samples were microbiologically analyzed per trial (*n* = 4).

### 2.2. Determination of Nutritional Parameters (Protein, Fat, Fatty Acid Profile, Carbohydrates, Moisture and Ash)

Analyses for the determination of protein, fat, carbohydrate, moisture and ash content in fresh and dried products were performed on the day of arrival at the laboratory.

The nitrogen content was calculated by the Kjeldahl method according to the procedure described in ISO 1871:2009 [21]. The protein content was estimated by multiplying the nitrogen content by five, as this conversion factor was previously suggested for the estimation of protein content of seaweeds [22,23]. The fat content was measured using Soxhlet extraction after acid hydrolysis (based on ISO 1443:1973 [24], ISO 1444:1996 [25]) while the fatty acid profile was determined by means of Gas Chromatography Flame Ionization Detector (GC-FID) following the procedure suggested by ISO 12966 [26]. The amount of carbohydrates was calculated indirectly by subtracting all the other constituents in the seaweeds (protein, fat, water, ash) from the total weight of the seaweeds. For the determination of the ash content, a quantity of 2–3 g of dried product was weighed in a tared crucible. The crucible was placed in a furnace and burned at 500 °C overnight. The crucibles were then transferred to a desiccator, allowed to cool and weighed. The difference between the weight after ashing and the tared weight of the crucible divided with the original sample weight represents the ash content. The water content was determined by initially drying porcelain containers containing sand for 2–3 h at 100 °C. They were then allowed to cool in a desiccator and weighed. A quantity of 2–3 g of sample was weighed into the container and mixed with the sand. Subsequently, they were dried in the oven at 100 °C overnight, allowed to cool in a desiccator and weighed [27]. For the determination of the sodium chloride content, 25 g of sample were weighed into a 400 mL beaker. Next, 200 mL of hot water were added and stirred for 60 min. The homogenate was then filtered, while the filtrate was collected in a 250 mL volumetric flask, made up to the volume and homogenized well. Ten milliliters of the filtrate were transferred into a 100 mL flask along with 50 mL of distilled water and 1 mL potassium chromate (K_2_CrO_4_) indicator. The amount of sodium chloride present in seaweeds was determined by titrating the extract containing the chloride ion with silver nitrate, AgNO_3_ (0.1 N). At the end point, the colour changed from yellow to brownish red.

### 2.3. Microbiological Analysis

Seaweed samples (15 g) were transferred aseptically to a stomacher bag and diluted ten times in sterile Maximum Recovery Diluent (MRD). The mixture was homogenized in a stomacher (Lab Blender, Seward Ltd., London, UK) for 120 s at room temperature. The resulting suspension was serially diluted in the same diluent and aliquots (0.1 or 1 mL) of the appropriate dilutions were spread or poured in duplicate in the following agar media: Marine Agar (Condalab, Madrid, Spain) for total viable counts (TVC), incubated at 30 °C for 48 h; Pseudomonas agar base (supplemented with cephalothin, fucidin, cetrimide selective supplement, LABM, Heywood, UK) for *Pseudomonas* spp., incubated at 25 °C for 48 h; Rose-Bengal Chloramphenicol Agar Base (supplemented with chloramphenicol, LABM) for yeasts and molds, incubated at 25 °C for 72 h; Violet Red Bile Glucose Agar (Biolife, Milan, Italy) for bacteria of Enterobacteriaceae family, overlaid with the same medium and incubated at 37 °C for 24 h; Mannitol Egg Yolk Polymyxin agar (Neogen, Lansing, MI, USA) supplemented with egg yolk emulsion 50% and polymyxin B for *Bacillus* spp., incubated at 35 °C for 40 h. After incubation, typical colonies for each microbial group were enumerated and colony counts were logarithmically transformed (log CFU/g). Results are presented as mean values (±standard deviation) of the four samples analyzed at each sampling point. The biomass was also examined for the presence of pathogenic bacteria. The presence of *Salmonella* was investigated by the process of selective enrichment by suspending 25 g of seaweed (fresh or dried) in 225 mL of Buffered Peptone Water (LABM, UK) incubated at 37 °C for 24 h (primary enrichment). Further on, a sample aliquot of 0.1 mL was transferred to 10 mL of Rappaport-Vassiliadis selective enrichment broth (LABM, UK) and incubated at 43 °C for 48 h (secondary enrichment). After each enrichment step, the culture was streaked onto XLD agar plates (LABM, UK) and incubated at 37 °C for 24 h. In the case of *Listeria monocytogenes*, 25 g of seaweed were suspended in 225 mL of Half Fraser Broth (Neogen, Lansing, MI, USA) at 30 °C for 48 h. After 24 h of incubation, 0.1 mL from the primary enrichment culture was transferred to 10 mL of Fraser Broth (Neogen, Lansing, MI, USA) and incubated at 37 °C for 48 h. After each of the aforementioned enrichment steps, the culture was streaked onto Listeria Palcam Agar Base (Biolife, Milan, Italy) and ALOA plates (Biolife, Milan, Italy) (incubation at 37 °C for 48 h). For confirmation of the results, a catalase test (on Tryptone Soya Yeast Extract), hemolysis test, carbohydrate utilization and CAMP test were performed. For the detection of *Vibrio* spp., 25 g of seaweed were added to Alkaline Peptone Water (pH 8.0—incubation 37 °C for 6 to 24 h). A small quantity of the culture (after 6 h and 24 h) was streaked onto TCBS Kobayashi Agar (Biolife, Milan, Italy), and incubated at 37 °C for 24 h. Typical *Vibrio* colonies were subsequently picked and streaked onto a non-selective agar (Marine Agar) for purity (incubation overnight at 37 °C). Single colonies were isolated and subjected to DNA extraction as described below (Section 2.4). A multiplex PCR method was employed for the detection of pathogenic *Vibrio* species; *V. alginolyticus*, *V. parahaemolyticus*, *V. vulnificus* and *V. cholerae* according to the method previously described [28].

### 2.4. Identification of Bacterial Species

Colonies (10–20%) developed on Marine Agar medium were randomly selected from the highest dilution and purified by successive subculture on the same medium at 30 °C. In total, 88 colonies from *A. esculenta* and 47 colonies from *S. latissima*, collected throughout the storage period, were isolated and purified. Purified bacteria colonies were subjected to Gram staining, oxidase test and catalase test, while colony appearance and cell morphology were also recorded. Pure bacterial cultures were stored at −20 °C in Marine Broth supplemented with 20% glycerol until needed. Bacterial DNA was obtained by a bacterial cell extraction method based on lysozyme, as previously described [29]. The total amount of nucleic acids extracted from samples was finally re-suspended in 25 μL DNase-free water. Quantification of total DNA and a quality check were carried out with a NanoDrop spectrophotometer (Implen, Munich, Germany). The DNA extracts were stored at −20 °C. To detect the bacterial diversity among the isolated colonies, a PCR-based DNA fingerprinting method was employed. Randomly amplified polymorphic DNA (RAPD)-PCR was first employed to all bacterial isolates, so as to create clusters of bacterial species. Amplification primer M13 universal primer (5′-GAGGGTGGCGGTTCT-3′) was used. PCR reaction mixture contained the following in a total volume of 50 μL: PCR-buffer (10 × PCR buffer Β with 1.5 mM MgCl_2_, Kapa Biosystems, Wilmington, MA, USA), additional 0.2 mM MgCl_2_, 0.8 mM dNTPs, 4 μM primer M13, 1 U Taq DNA polymerase (Kapa Biosystems, USA), DNA (100 ng) and sterile distilled water. PCR amplification was performed under the following conditions: an initial denaturation step at 95 °C for 3 min, 3 cycles of denaturation at 95 °C for 3 min, primer annealing at 35 °C for 5 min and primer elongation 72 °C for 5 min, followed by 32 cycles of denaturation at 95 °C for 1 min, primer annealing at 55 °C for 2 min and primer elongation 72 °C for 3 min, and a final elongation step at 72 °C for 7 min. Separation of PCR products by electrophoresis was performed on a 1.5% agarose gel in 1 × TAE (40 mM Tris–acetate, 1 mM EDTA, pH 8.2) buffer at 100 V for 75 min. Gels were stained with ethidium bromide and visualized under UV light in a Bio-Rad GelDoc 2000 system (Bio-Rad Laboratories Inc., Hercules, CA, USA) using the analysis software Quantity-One (Bio-Rad, Hercules, CA, USA). Gel images were edited appropriately and analyzed using the Jaccard/Dice coefficient and the unweighted pair group method with arithmetic mean (UPGMA) cluster analysis, by using the BioNumerics software version 6.1 (Applied Maths, Sint-Martens-Latem, Belgium). Representative strains of distinct RAPD-PCR clusters were selected and subjected to species identification by partial sequencing of the 16S rRNA gene, targeting the hypervariable regions V1–V3, as previously described [30]. PCR amplification products were purified using the NucleoSpin^®^ Gel and PCR Clean-up (Macherey-Nagel, Dueren, Germany) following the manufacturer’s instructions. Sequencing was performed by CeMIA SA (Larissa, Greece). Sequencing data were aligned to the closest relative in the database using the BLAST algorithm optimized for highly similar sequences (Blastn) (www.ncbi.nlm.nih.gov/blast (accessed on 15 March 2021)). Sequences with 97% or higher identity were considered to represent the same species.

### 2.5. Statistical Analysis

All data are presented as mean values ± standard deviation. The effect of storage time and drying temperature on the microbial counts as well as the differences in nutritional parameters across species and year were evaluated using one-way analysis of variance (ANOVA), while significant differences among cases (*p* < 0.05) were determined by Tukey’s HSD test (XLSTAT software version 2012.04.1 (Addinsoft, Paris, France)).

## 3. Results and Discussion

### 3.1. Nutritional Analysis

Nutritional parameter values differed across species, year of harvest and sample condition (Table 2). The protein content of either species was significantly higher in 2019 than in 2020, with 2.44 vs. 1.99 g/100 g and 1.40 vs. 0.87 g/100 g for fresh *A. esculenta* and *S. latissima*, respectively, with higher values in 2019 likely being explained by the more evident biofouling cover observed in this batch. Similar to the fresh samples, the protein content of dried samples also differed between the two years, whilst in both years significantly higher levels of protein were observed in *A. esculenta* compared to *S. latissima*, being in line with values reported in the literature [6,31]. Although brown seaweeds are found to be lower in protein compared to red species, they are characterized by the presence of almost all essential amino acids needed for both humans and animals [32].

In dried *A. esculenta* samples, the total carbohydrate content ranged from 50.7 to 53.3 g/100 g, compared to *S. latissima* ranging from 42.1 to 43.9 g/100 g for 2019 and 2020, respectively. Previous studies have reported similar levels of carbohydrates, which are mainly composed of alginates and secondarily of mannitol, glucose and fucose [6,31,33].

The ash content is an important parameter of seaweed nutritional quality as it is related to minerals and trace elements. The ash values in fresh samples were not significantly differentiated across species and year of harvest. On the other hand, the ash content of dried *S. latissima* was significantly higher compared to *A. esculenta* in both years (26.00–26.34 vs. 37.66–38.94 g/100 g, for *A. esculenta* and *S. latissima*, respectively). In addition, the NaCl content, which is included in the ash content, was also increased in dried *S. latissima* samples (24–28 g/100 g DW) compared to that of *A. esculenta* (15–16 g/100 g DW), although previous studies have proposed *S. latissima* as a salt-replacing ingredient due to its low Na/K ratio [4,34].

The total fat content in the dried samples of both species was low (0.55–0.82 g/100 g DW). However, despite the low total lipid concentration, they contain significant amounts of essential mono- and poly-unsaturated fatty acids and offer many beneficial effects on human health [35].

### 3.2. Microbial Profile of Fresh Seaweeds

**Figure 1:**
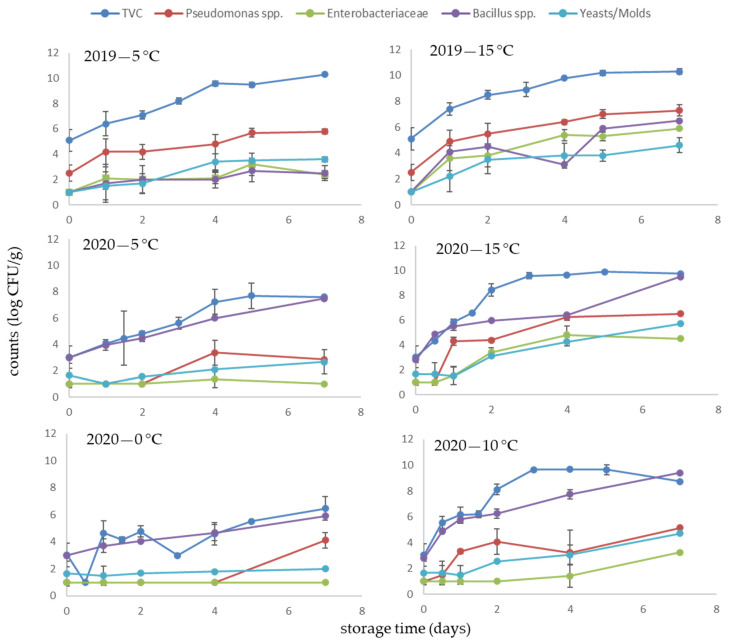
Changes in the microbial counts of *A. esculenta* stored aerobically at 5 and 15 °C (year 2019) and 0, 5, 10 and 15 °C (year 2020).

The changes in the microbial populations of fresh *A. esculenta* samples throughout storage for the two harvest years are presented in Figure 1. In general, microbial counts in 2020 samples were remarkably lower compared to 2019. The initial TVC counts were 3.2 and 5.2 log CFU/g in 2020 and 2019 samples, respectively, while microorganisms reached the level of 7.0 log CFU/g on days 2 and 4 at 5 °C in 2019 and 2020 samples, respectively. Previous studies have also reported 7.0–8.0 log CFU/g as the threshold level for the onset of deterioration in marine algae [19,20]. For the 2019 samples, *Pseudomonas* spp. dominated the observed microbial groups at both storage temperatures. The specific spoilage microorganisms in the 2020 samples, *Bacillus* spp. and *Pseudomonas* spp., were found at levels similar to TVC, particularly at lower storage temperatures (0 and 4 °C). It should be noted that low levels of Enterobacteriaceae were detected, especially in the 2020 samples, indicating good hygiene practices [36].

Colonies isolated from Marine Agar (2020 samples only) were further examined to elucidate the microbial diversity of the seaweeds tested. For *A. esculenta*, thirteen different bacterial species were identified, mainly belonging to the genera *Psychrobacter*, *Cobetia* and *Pseudomonas* (Table 3). More specifically, the major species identified were *Cobetia crustatorum* (19 fingerprints), *Psychrobacter fozii* (15 fingerprints), *Pseudomonas psychrophila* (11 fingerprints), *Psychrobacter adeliensis* (10 fingerprints), *Psychrobacter piscatorii* (6 fingerprints) and *Lelliottia amnigena* (5 fingerprints). *C. crustatorum* and *P. fozii* were present throughout storage at 0 and 10 °C, while the presence of pseudomonads was limited to seaweeds stored at 15 °C. Fingerprint data and clustering are shown in Supplementary Material (Figure S1).

**Figure 2:**
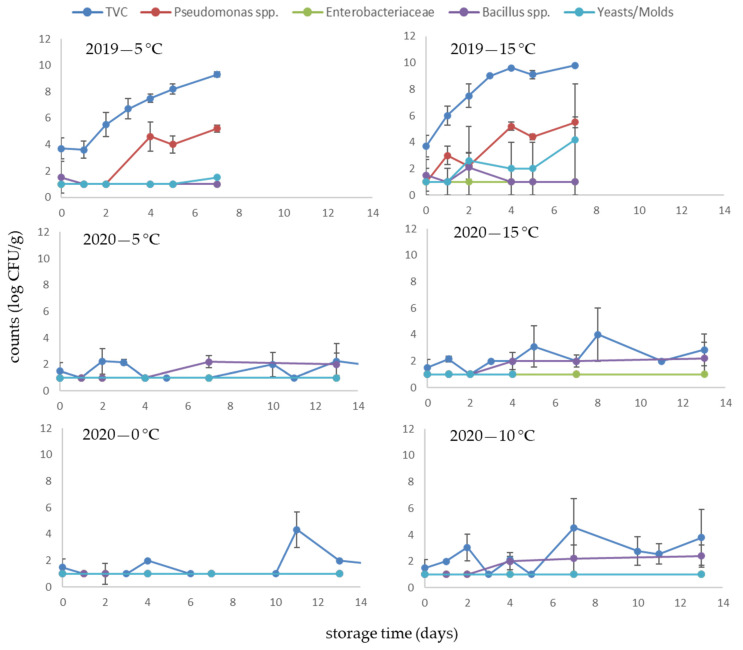
Changes in the microbial counts of *S. latissima* stored aerobically at 5 and 15 °C (year 2019) and 0, 5, 10 and 15 °C (year 2020).

In the case of fresh *S. latissima*, the initial microbial counts were lower compared to *A. esculenta*, even though they were cultivated in close proximity to each other. This difference could be attributed to the different morphology of the two species. *A. esculenta* usually has longer fronds which grow faster, but it is more prone to biofouling compared to *S. latissima* [37]. It has been also reported that different microbial communities are found in different species of seaweeds, even if they grow in the same ecological niche [38,39]. Similar to *A. esculenta*, the microbial evolution in *S. latissima* samples harvested in 2020 was significantly different from that of 2019 (Figure 2). For the 2019 samples, the initial TVC counts were 3.7 log CFU/g, while the level of 7–8 log CFU/g was reached on the 4th and 2nd day of storage at 5 and 15 °C, respectively. In 2020 samples, no specific growth pattern was observed, while at low temperatures the microbial population remained close to the detection limit (1.0 log CFU/g) even after a long storage period (60 days at 0 °C). At higher temperatures (10 and 15 °C), some fluctuations in microbial counts were only observed throughout storage, while all microbial groups were close to or below the detection limit (1.0 log CFU/g).

Molecular analysis of the isolated colonies from Marine Agar (for 2020 samples) revealed 12 different bacterial species belonging to the genera *Psychrobacter*, *Micrococcus*, and *Staphylococcus* (Table 4). The majority of the identified species were *P. fozii* (9 fingerprints), *Micrococcus luteus* (6 fingerprints) and *Psychrobacter pacificensis* (5 fingerprints). Moreover, *B. cereus* was identified in samples from the late storage period at 5 °C. Bacteria of the *Micrococcus* genus were mainly isolated from fresh samples, whereas *Bacillus* and *Psychrobacter* species were recovered from samples of late storage at 5 and 15 °C, respectively. In addition, storage temperature influenced the diversity of the culturable bacteria isolated; at 5 °C eleven different bacterial species were identified, whereas at 15 °C only three species were isolated (Table 4). Fingerprint data and clustering are shown in Supplementary Material (Figure S2).

Bacteria of *Staphylococcus* spp. were isolated in the samples of *S. latissima* at the beginning of storage, which were probably transmitted by hand during harvest and handling operations.

In general, the microbial load of fresh brown seaweeds varies from 1.0 to 7.0 log CFU/g [14,17,40]. Many factors affect this population range, including species differentiation, location, environmental conditions and seasonal differentiation, as well as the seaweed life cycle and the fact that different bacterial communities are present on different parts of the seaweed thallus [15,20]. Seaweed quality assessment is the main focus of recent studies, as various factors including chemical, enzymatic and microbiological alterations contribute to the quality degradation of these products [19]. Although there are few studies investigating the microbial diversity of marine algae, and even fewer estimating the microbial growth and the shelf life of different seaweed species, almost none have focused on the spoilage potential of the identified microorganisms and the type of deterioration they cause. In our study, the main microbial groups identified in *A. esculenta*, belonged to the genera *Psychrobacter*, *Cobetia*, and *Pseudomonas*, while in *S. latissima*, bacteria of *Psychrobacter* and *Micrococcus* spp. genera were identified. Additionally, the presence of *Bacillus* spp. was confirmed by both molecular and conventional microbiological analysis. The abundance of *Psychrobacter* species has been reported previously in seaweeds including *Ulva lactuca*, *Undaria pinatifada*, *Laminaria ochroleuca*, *Palmaria palmata*, etc. [15,41]. Some strains of this bacterium have shown a wide range of enzymatic activity, breaking down short- to medium-chain lipids and hydrolyzing amino acids (leucine), affecting the quality of the end-product [42,43]. However, it is considered as a moderate spoiler, since many of the strains of this genus produce weak off-flavors lacking important spoilage potential, such as proteolysis and sulphide production [44]. *Cobetia* spp. has been also isolated from several seaweed species [15,45]. Most strains require NaCl up to 5% for optimum growth [46] while some present polysaccharide-degrading activity [47]. *Pseudomonas* spp. has been reported as a typical spoiler of seafood products such as finfish and shellfish, producing malodorous compounds. Moreover, pseudomonads can negatively affect the quality of leafy vegetables—which are similar to seaweed tissues—due to the pectinolytic activity of some species that can cause soft rot of green leafy vegetables and consequently decrease the shelf life [48,49,50].

The isolation of potentially toxin-producing spore-forming bacteria of the *Bacillus* genus from *A. esculenta* and *S. latissima* [17], as well as from other brown seaweed species [15,51], has been previously confirmed. Apart from toxin production that can be threatening for human health, *Bacillus* spp. could also contribute to soft rot of plant and seaweed tissues, resulting in quality degradation [49]. Finally, micrococci that were found in *S. latissima* could also produce off-odors and negatively affect seafood quality [52]. Apart from the contribution of the identified microorganisms to the seaweeds’ degradation, the high microbial diversity between the two species should also be underlined. Although they were cultivated in proximity to each other, and harvested and processed similarly, concerning the fresh samples, the culturable microbial communities were comprised of different microorganisms in each seaweed species, indicating the impact of species on the microbial profile and subsequently on the spoilage potential of each macroalgae.

Concerning the prevalence of specific pathogens related to human illness, *Salmonella*, *E. coli*, and *Staphylococcus aureus* were absent in seaweed samples from both years, while *L. monocytogenes* was found in one *A. esculenta* sample from 2019, probably due to cross contamination after harvest. Bacteria of the genus *Vibrio* were also isolated from *A. esculenta* samples of both years. None of the twelve isolates belonged to *V. vulnificus*, *V. parahaemolyticus* or *V. cholerae* species, while two of them identified as *V. alginolyticus*. *V. alginolyticus* is one of the most common pathogenic *Vibrio* species and has been found to cause serious infections in both humans and animals [53]. In humans, soft tissues, the ear and superficial wounds are easily invaded by *V. alginolyticus*, while in animals, septicemia, melanosis, white spot syndrome and necrosis are among the most common diseases caused by this microorganism, resulting in increased mortality rates [54]. Regarding *S. latissima*, none of the aforementioned pathogenic bacteria was detected.

### 3.3. Microbiological Quality of Dried Products

Seaweeds are considered as highly perishable foodstuff due to the high moisture and nutrient content, and thus drying is necessary to reduce water activity (a_w_) and increase the shelf life of the product [40,55,56]. Most published studies have focused on the effect of drying conditions on the nutritional quality or on several physicochemical and sensory characteristics [10,57,58].

**Figure 3:**
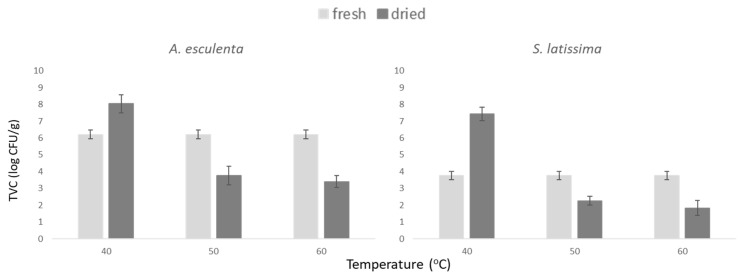
Effect of different drying temperatures on total aerobe (TVC) populations in seaweeds *A. esculenta* and *S. latissima*.

To determine the most appropriate temperature conditions that should be applied in the drying process, three temperatures (40, 50 and 60 °C) were tested on a lab-scale, taking into consideration the need for mild processing conditions in order to maintain nutritional quality and also promote sustainable practices (Figure 3). Previous studies have reported that 40 °C was the optimum temperature in terms of nutritional quality [40], and in general, the current trend supports drying temperatures not exceeding 50 °C [12]. Although 40 °C could be an ideal temperature for maintaining the nutritional quality of seaweeds, it was found to be inadequate from the microbiological perspective, as a 2-log increase was observed in TVC counts in both seaweed species. On the other hand, no particular differentiation was observed between drying at 50 and 60 °C, as at both temperatures TVC counts decreased by 2.0 log CFU/g. Based on this observation, 50 °C could be considered an appropriate temperature to produce seaweed of high microbiological and nutritional quality.

**Figure 4:**
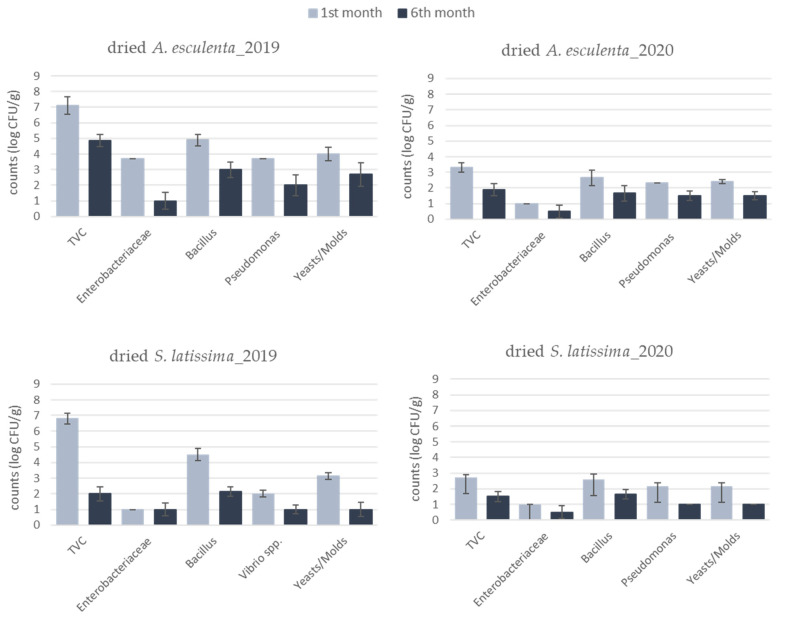
Microbiological profile of dried seaweeds *A. esculenta* and *S. latissima* harvested in 2019 and 2020, after 1 and 6 months of storage at 22 °C.

The dried products of 2020 were also quite different compared to 2019 (Figure 4). Specifically, in 2019 samples, the microbial counts reached 7.0 log CFU/g in both seaweed species, indicating a foodstuff of questionable microbiological quality that is probably inappropriate for human consumption. In the case of *A. esculenta*, bacteria of the Enterobacteriaceae family were also detected at increased levels (close to 4.0 log CFU/g). On the other hand, in 2020 samples, the microbiological quality was noticeably improved, with TVC counts being lower than 3.0 log CFU/g in both species. It needs to be noted that in both years microbial counts decreased after 6 months of storage, probably due to moisture loss and high NaCl content resulting in a progressive loss of the microorganisms’ viability. Del Olmo et al. (2018) [41] investigated the microbiological quality of several dried seaweed species. The microbial population presented high variability (ranging from 1.5 to 7.5 log CFU/g) among different seaweed species and also among different batches of the same species. In this work, the level of TVC in *S. latissima* was 3.1 log CFU/g on average (range: 2.0–4.2 log CFU/g). Additionally, *Bacillus* spp. was detected at relatively high populations (4.5–5.0 log CFU/g) in dried samples of both species in 2019. This microbial group includes spore-forming bacteria that could survive after thermal processing and may pose a risk to consumers’ health. Previous studies have also reported a high incidence of *Bacillus* spp. on seaweeds due to its ability to survive under stressful conditions and, particularly, at high NaCl (>10%) concentrations [41,59]. Finally, pathogenic *Vibrio* spp., *Salmonella*, *E. coli*, *L. monocytogenes* and *Staphylococcus aureus* were absent in seaweed samples from both years. Factors that may affect the quality of dried seaweed products include the initial microbial load, drying conditions, post drying handling of the samples (cross contamination could be a significant risk in such products before packaging) and the quality of the packaging material. Consequently, the differences observed could be attributed to the different drying procedures employed in this work (indoor and outdoor drying) and the prevailing environmental conditions in both years.

### 3.4. Rehydrated Products

**Figure 5:**
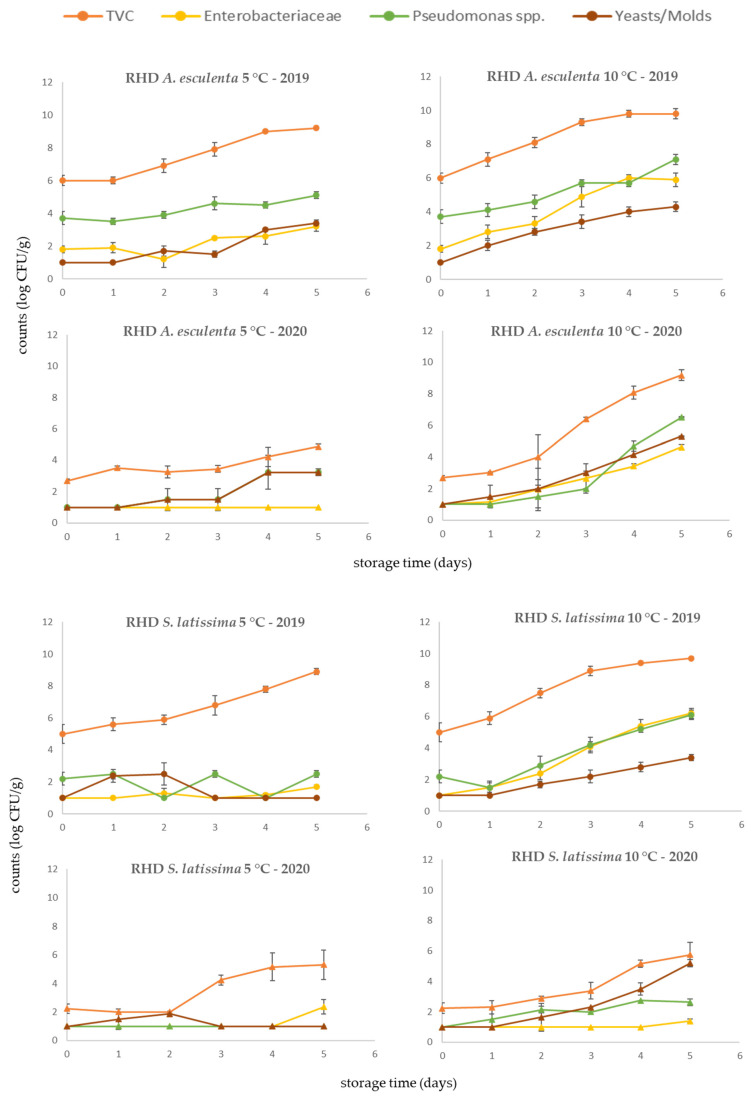
Microbial populations of rehydrated (RHD) seaweeds *A. esculenta* and *S. latissima* in 2019 and 2020 harvest years, stored at 5 and 10 °C.

The microbial populations of rehydrated products are presented in Figure 5. Microbial counts followed the same pattern with the fresh and dried seaweed samples as mentioned previously. The initial TVC counts were similar to that of the dried products used in their preparation. In both harvest years and seaweed species, the initial microbial load of rehydrated seaweeds was 1.0 log CFU/g higher compared to the dried samples. This difference could be attributed to rehydration given that the moisture content of dried samples was ca. 10%. In the case of *A. esculenta,* the low initial microbial load in the samples of 2020 had an impact on the shelf life of the product and the maintenance of high microbiological quality throughout storage, especially in refrigerated conditions (4 °C). In *S. latissima* samples, microorganisms were at low levels after five days of storage even at 10 °C.

The rehydration of dried seaweeds is considered a common household practice aiming to restore the properties of the fresh product. Although it was not considered in the present study, water temperature is the most important factor influencing rehydration. In general, higher rehydration rates are obtained at higher water temperatures, despite the fact that high water temperatures may result in significant losses of important phenolic compounds and nutrients [60]. Apart from the nutritional and physicochemical quality, rehydration may negatively affect the microbiological profile, promoting microbial growth including pathogenic species. In order to minimize this effect, the high quality level of the initial dried seaweeds should be ensured, together with good hygiene practices during handling to avoid cross-contamination.

## 4. Conclusions

The microbial load of two seaweed species (*A. esculenta* and *S. latissima*) differed between two harvest years (2019 and 2020), and the contributing factors should be further investigated. Additionally, emphasis should be given to the diverse microbial profile between the two species, considering that they were grown in proximity to each other and were handled in a similar way. Moreover, the initial microbial load of the fresh samples was found to be a critical parameter for the quality of dried and rehydrated products, whilst the quality of dried products was strongly affected by the quality of the raw material as well as the drying conditions. This study showed that drying and handling procedures do affect the microbiological quality of seaweeds and thus need to be considered when assessing seaweed products. Taking the aforementioned into account, seaweeds—under certain circumstances—could be considered as a highly perishable foodstuff. Therefore, it is important for seaweed producers and retailers to be aware of this and proceed promptly with further processing (freezing, drying, etc.) or marketing of the fresh products.

## Figures and Tables

**Table 1:** Brief description of the experimental design.

**A**	***Alaria esculenta***					
**Harvest year**	**2019**			**2020**		
**Form**	**Fresh/Frozen**	**Dried**	**Rehydrated**	**Fresh/Frozen**	**Dried**	**Rehydrated**
Storage temperatures (°C)	5, 15	25	5, 10	0, 5, 10, 15	25	5, 10
Storage time	7 days	6 months	5 days	7 days	6 months	5 days
Microbiological analysis	7 time points	2 time points	6 time points	9 time points	2 time points	6 time points
Number of replicates	4 *	4	4	4	4	4
Nutritional analysis	Day 0	Day 0	-	Day 0	Day 0	-
Number of replicates	3	3	-	3	3	-
**B**	***Saccharina latissima***					
**Harvest year**	**2019**			**2020**		
**Form**	**Fresh/Frozen**	**Dried**	**Rehydrated**	**Fresh/Frozen**	**Dried**	**Rehydrated**
Storage temperatures (°C)	5, 15	25	5, 10	0, 5, 10, 15	25	5, 10
Storage time	7 days	6 months	5 days	60 (0 °C), 25 (5 °C, 13 days (at 10 and 15 °C)	6 months	5 days
Microbiological analysis	7 time points	2 time points	6 time points	8 time points (0, 5 °C), 9 time points (10, 15 °C)	2 time points	6 time points
Number of replicates	4	4	4	4	4	4
Nutritional analysis	Day 0	Day 0	-	Day 0	Day 0	-
Number of replicates	3	3	-	3	3	-

* samples/time point/temperature.

**Table 2:** Nutritional parameters of fresh and dried seaweeds *A. esculenta* and *S. latissima* in 2019 and 2020 harvest years.

g/100 g	*A. esculenta*				*S. latissima*			
	2019		2020		2019		2020	
	Fresh	Dried	Fresh	Dried	Fresh	Dried	Fresh	Dried
Protein	2.44 ± 0.10 ^a^ *	11.30 ± 0.06 ^A^ *	1.99 ± 0.08 ^a^ *	9.13 ± 0.35 ^A^ *	1.40 ± 0.14 ^a^ *	9.93 ± 0.14 ^A^ *	0.87 ± 0.01 ^b^ *	7.44 ± 0.12 ^B^ *
Fat	0.21 ± 0.02 ^a^ *	0.82 ± 0.10 ^A^ *	0.13 ± 0.08 ^a^	0.60 ± 0.09 ^A^	0.11 ± 0.06 ^a^ *	0.55 ± 0.08 ^A^ *	0.08 ± 0.01 ^a^	0.73 ± 0.10 ^A^
Content (%)								
Saturated	-	49.53 ± 1.80	-	73.97 ± 1.00	-	62.72 ± 1.30	-	79.91 ± 1.00
MUFA	-	26.67 ± 1.50	-	13.60 ± 0.80	-	24.44 ± 1.00	-	8.74 ± 0.10
PUFA	-	23.79 ± 0.96	-	12.43 ± 0.94	-	12.85 ± 0.91	-	11.35 ± 0.70
Carbohydrates	14.64 ± 1.00 ^a^ *	50.72 ± 1.00 ^A^ *	14.41 ± 0.90 ^a^ *	53.28 ± 1.20 ^A^ *	6.93 ± 1.00 ^a^ *	42.16 ± 1.42 ^A^ *	6.91 ± 0.91 ^a^ *	43.97 ± 1.00 ^B^ *
Moisture	78.83 ± 1.20 ^a^ *	11.16 ± 0.74 ^A^	80.55 ± 1.30 ^a^ *	10.64 ± 0.01 ^A^	88.30 ± 1.60 ^a^ *	9.70 ± 0.95 ^A^	89.40 ± 0.50 ^a^ *	8.92 ± 0.50 ^A^
Ash	3.88 ± 0.64 ^a^	26.00 ± 1.40 ^A^ *	2.92 ± 0.18 ^a^	26.34 ± 0.78 ^A^ *	3.26 ± 0.98 ^a^	37.66 ± 1.30 ^A^ *	2.74 ± 0.17 ^a^	38.94 ± 1.90 ^A^ *
NaCl	2.00 ± 0.40 ^a^	15.00 ± 0.58 ^A^ *	1.80 ± 0.10 ^a^	16.00 ± 1.00 ^A^ *	2.00 ± 0.64 ^a^	28.00 ± 0.50 ^A^ *	1.85 ± 0.10 ^a^	24.00 ± 0.80 ^B^ *

Data are represented as means ± standard deviation (*n* = 3). MUFA: monounsaturated fatty acids, PUFA: polyunsaturated fatty acids. Different letters indicate significant differences (*p* < 0.05) among cases (Uppercase letters: dried samples (of the same species), lowercase letters: fresh samples (of the same species)), asterisk *: significant differences between species (of the same year and condition).

**Table 3:** Species identification of bacteria isolated from *A. esculenta* (SAE).

Isolate Code	Temperature °C	Days of Storage	TVC (Log CFU/g)	Closest Relative Microorganism	GenBank Accession Number of Closest Relative	% Similarity
SAE18	5	3	5.6	*Cobetia crustatorum*	NR_116500.1	99.13
SAE19				*Psychrobacter fozii*	NR_025531.1	98.30
SAE20				*Pseudoalteromonas tetraodonis GFC*	NR_119142.1	98.08
SAE37	5	5	7.7	*Psychrobacter fozii*	NR_025531.1	99.83
SAE40				*Cobetia crustatorum*	NR_116500.1	99.31
SAE60	5	7	8.0	*Corynebacterium tapiri*	NR_145582.1	95.81
SAE61				*Lelliottia amnigena*	NR_024642.1	99.83
SAE62				*Jeotgalicoccus psychrophilus*	NR_025644.1	99.14
SAE67				*Cobetia litoralis*	NR_113403.1	86.68
SAE69				*Psychrobacter cryohalolentis K5*	NR_075055.1	99.30
SAE70				*Psychrobacter fozii*	NR_025531.1	100.0
SAE72				*Cobetia crustatorum*	NR_116500.1	99.13
SAE03	15	1	6.0	*Psychrobacter piscatorii*	NR_112807.1	100.0
SAE09				*Psychrobacter fozii*	NR_025531.1	100.0
SAE10				*Cobetia crustatorum*	NR_116500.1	99.13
SAE22	15	3	9.5	*Cobetia crustatorum*	NR_116500.1	99.30
SAE23				*Pseudomonas psychrophila*	NR_028619.1	99.65
SAE36				*Pseudomonas weihenstephanensis*	NR_148764.1	99.65
SAE50	15	5	10.0	*Lelliottia amnigena*	NR_024642.1	99.83
SAE51				*Pseudomonas psychrophila*	NR_028619.1	99.65
SAE59				*Pseudomonas monteilii*	NR_112073.1	99.48
SAE75	15	7	9.5	*Psychrobacter adeliensis*	NR_117632.1	93.82
SAE76				*Psychrobacter fozii*	NR_025531.1	100.0
SAE85				*Pseudomonas weihenstephanensis*	NR_148764.1	99.65

**Table 4:** Species identification of bacteria isolated from *S. latissima* (SSL).

Isolate Code	Temperature °C	Days of Storage	TVC (Log CFU/g)	Closest Relative Microorganism	GenBank Accession Number of Closest Relative	% Similarity
SSL01	5	0	1.5	*Mesobacillus subterraneus*	MT515815.1	99.67
SSL03				*Micrococcus luteus*	NR_037113.1	99.48
SSL06				*Staphylococcus hominis*	NR_036956.1	94.55
SSL10				*Staphylococcus epidermidis*	NR_036904.1	99.83
SSL11	5	2	2.2	*Micrococcus aloeverae*	NR_134088.1	99.10
SSL12				*Acinetobacter* *i* *woffii*	NR_113346.1	100.0
SSL14				*Roseomonas aestuarii*	NR_114285.1	100.0
SSL18	5	3	2.0	*Micrococcus luteus*	NR_037113.1	99.82
SSL43	5	13	2.2	*Alkalihalobacillus hwajinpoensis*	NR_025264.1	96.14
SSL47				*Bacillus cereus*	R_115526.1	100.0
SSL24	15	7	2.0	*Lelliottia amnigena*	NR_024642.1	99.83
SSL26				*Psychrobacter pacificensis*	NR_027187.1	100.0
SSL30	15	8	4.0	*Psychrobacter fozii*	NR_025531.1	99.65

## Data Availability

The data presented in this study are available on request from the corresponding author.

## Supplementary Materials

The following are available online at https://www.mdpi.com/article/10.3390/foods10092210/s1, Figure S1: RAPD-PCR clustering of bacteria isolated from *A. esculenta* (fingerprints analyzed with Dice coefficient and dendrogram constructed with UPGMA), Figure S2: RAPD-PCR clustering of bacteria isolated from *S. latissima* (fingerprints analyzed with Dice coefficient and dendrogram constructed with UPGMA).

## References

[1] Ferdouse F., Holdt S.L., Smith R., Murúa P., Yang Z., FAO Consultants (2018). The Global Status of Seaweed Production, Trade and Utilization.

[2] Delaney A., Frangoudes K., Li S.A., Levine I., Fleurence J. (2016). Society and seaweed: Understanding the past and present. Seaweed in Health and Disease Prevention.

[3] Mouritsen O.G., Rhatigan P., Pérez-Lloréns J.L. (2018). The rise of seaweed gastronomy: Phycogastronomy. Bot. Mar..

[4] Rioux L.-E., Beaulieu L., Turgeon S.L. (2017). Seaweeds: A traditional ingredients for new gastronomic sensation. Food Hydrocoll..

[5] Perry J.J., Brodt A., Skonberg D.I. (2019). Influence of dry salting on quality attributes of farmed kelp (*Alaria esculenta*) during long-term refrigerated storage. LWT.

[6] Stévant P., Marfaing H., Rustad T., Sandbakken I., Fleurence J., Chapman A. (2017). Nutritional value of the kelps *Alaria esculenta* and *Saccharina latissima* and effects of short-term storage on biomass quality. Environ. Boil. Fishes.

[7] Handå A., Forbord S., Wang X., Broch O.J., Dahle S.W., Størseth T.R., Reitan K.I., Olsen Y., Skjermo J. (2013). Seasonal and depth-dependent growth of cultivated kelp (*Saccharina latissima*) in close proximity to salmon (*Salmo salar*) aquaculture in Norway. Aquaculture.

[8] Kraan S., Tramullas A.V., Guiry M.D. (2000). The edible brown seaweed *Alaria esculenta* (*Phaeophyceae, Laminariales*): Hybridization, growth and genetic comparisons of six Irish populations. Environ. Boil. Fishes.

[9] Neto R.T., Marçal C., Queirós A.S., Abreu H., Silva A.M.S., Cardoso S.M. (2018). Screening of *Ulva rigida*, *Gracilaria* sp., *Fucus vesiculosus* and *Saccharina latissima* as functional ingredients. Int. J. Mol. Sci..

[10] Stévant P., Indergård E., Ólafsdóttir A., Marfaing H., Larssen W.E., Fleurence J., Roleda M., Rustad T., Slizyte R., Nordtvedt T. (2018). Effects of drying on the nutrient content and physico-chemical and sensory characteristics of the edible kelp *Saccharina latissima*. Environ. Boil. Fishes.

[11] Blikra M.J., Wang X., James P., Skipnes D. (2021). *Saccharina latissima* cultivated in northern Norway: Reduction of potentially toxic elements during processing in relation to cultivation depth. Foods.

[12] López-Pérez O., Picon A., Nuñez M. (2017). Volatile compounds and odour characteristics of seven species of dehydrated edible seaweeds. Food Res. Int..

[13] Banach J.L., Hil E.H., Van Der Fels-Klerx H.J. (2020). Food safety hazards in the European seaweed chain. Compr. Rev. Food Sci. Food Saf..

[14] del Olmo A., Picon A., Nuñez M. (2020). Preservation of five edible seaweeds by high pressure processing: Effect on microbiota, shelf life, colour, texture and antioxidant capacity. Algal Res..

[15] Singh R., Reddy C. (2014). Seaweed-microbial interactions: Key functions of seaweed-associated bacteria. FEMS Microbiol. Ecol..

[16] Egan S., Harder T., Burke C., Steinberg P., Kjelleberg S., Thomas T. (2013). The seaweed holobiont: Understanding seaweed-bacteria interactions. FEMS Microbiol. Rev..

[17] Blikra M., Løvdal T., Vaka M.R., Roiha I.S., Lunestad B.T., Lindseth C., Skipnes D. (2019). Assessment of food quality and microbial safety of brown macroalgae (*Alaria esculenta* and *Saccharina latissima*). J. Sci. Food Agric..

[18] Nayyar D., Skonberg D.I. (2018). Contrasting effects of two storage temperatures on the microbial, physicochemical, and sensory properties of two fresh red seaweeds, *Palmaria palmata* and *Gracilaria tikvahiae*. Environ. Boil. Fishes.

[19] Sánchez-García F., Hernández I., Palacios V.M., Roldán A.M. (2021). Freshness quality and shelf-life evaluation of the seaweed *Ulva rigida* through physical, chemical, microbiological, and sensory methods. Foods.

[20] Picon A., Del Olmo A., Nuñez M. (2020). Bacterial diversity in six species of fresh edible seaweeds submitted to high pressure processing and long-term refrigerated storage. Food Microbiol..

[21] ISO (2009). Food and Feed Products—General Guidelines for the Determination of Nitrogen by the Kjeldahl Method (1871:2009).

[22] Stévant P., Ólafsdóttir A., Déléris P., Dumay J., Fleurence J., Ingadóttir B., Jónsdóttir R., Ragueneau E., Rebours C., Rustad T. (2020). Semi-dry storage as a maturation process for improving the sensory characteristics of the edible red seaweed dulse (*Palmaria palmata)*. Algal Res..

[23] Angell A.R., Mata L., de Nys R., Paul N. (2015). The protein content of seaweeds: A universal nitrogen-to-protein conversion factor of five. Environ. Boil. Fishes.

[24] ISO (2016). Meat and Meat Products—Determination of Total Fat Content (1443:1973).

[25] ISO (2018). Meat and Meat Products—Determination of Free Fat Content (1444:1996).

[26] ISO (2017). Animal and Vegetable Fats and Oils—Gas Chromatography of Fatty Acid Methyl Esters—Part 2: Preparation of Methyl Esters of Fatty Acids (12966-2:2017).

[27] Nielsen S.S. (2010). Food analysis. Food Science Text Series.

[28] Wei S., Zhao H., Xian Y., Hussain M.A., Wu X. (2014). Multiplex PCR assays for the detection of *Vibrio alginolyticus*, *Vibrio parahaemolyticus*, *Vibrio vulnificus*, and *Vibrio cholerae* with an internal amplification control. Diagn. Microbiol. Infect. Dis..

[29] Doulgeraki A.I., Paramithiotis S., Nychas G.-J. (2011). Characterization of the Enterobacteriaceae Community that developed during storage of minced beef under aerobic or modified atmosphere packaging conditions. Int. J. Food Microbiol..

[30] Tzamourani A.P., Di Napoli E., Paramithiotis S., Oikonomou-Petrovits G., Panagiotidis S., Panagou E.Z. (2021). Microbiological and physicochemical characterisation of green table olives of Halkidiki and Conservolea varieties processed by the Spanish method on industrial scale. Int. J. Food Sci. Technol..

[31] Schiener P., Black K.D., Stanley M.S., Green D. (2014). The seasonal variation in the chemical composition of the kelp species *Laminaria digitata*, *Laminaria hyperborea*, *Saccharina latissima* and *Alaria esculenta*. Environ. Boil. Fishes.

[32] Mæhre H.K., Malde M.K., Eilertsen K.E., Elvevoll E. (2014). Characterization of protein, lipid and mineral contents in common Norwegian seaweeds and evaluation of their potential as food and feed. J. Sci. Food Agri..

[33] Holdt S.L., Kraan S. (2011). Bioactive compounds in seaweed: Functional food applications and legislation. Environ. Boil. Fishes.

[34] López-López I., Bastida S., Ruiz-Capillas C., Bravo L., Larrea M., Sánchez-Muniz F., Cofrades S., Jiménez-Colmenero F. (2009). Composition and antioxidant capacity of low-salt meat emulsion model systems containing edible seaweeds. Meat Sci..

[35] Rocha C., Pacheco D., Cotas J., Marques J., Pereira L., Gonçalves A. (2021). Seaweeds as valuable sources of essential fatty acids for human nutrition. Int. J. Environ. Res. Public Health.

[36] Biohaz E.P., Ricci A., Allende A., Bolton D., Chemaly M., Davies R., Escámez P.S.F., Girones R., Herman L., Koutsoumanis K. (2017). Guidance on the requirements for the development of microbiological criteria. EFSA J..

[37] Kerrison P.D., Innes M., Macleod A., McCormick E., Elbourne P.D., Stanley M.S., Hughes A.D., Kelly M.S. (2020). Comparing the effectiveness of twine and binder-seeding in the Laminariales species *Alaria esculenta* and *Saccharina latissima*. Environ. Boil. Fishes.

[38] Lachnit T., Blümel M., Imhoff J.F., Wahl M. (2009). Specific epibacterial communities on macroalgae: Phylogeny matters more than habitat. Aquat. Biol..

[39] Bondoso J., Balagué V., Gasol J.M., Lage O. (2014). Community composition of the Planctomycetes associated with different macroalgae. FEMS Microbiol. Ecol..

[40] Gupta S., Cox S., Abu-Ghannam N. (2011). Effect of different drying temperatures on the moisture and phytochemical constituents of edible Irish brown seaweed. LWT.

[41] Del Olmo A., Picon A., Nuñez M. (2018). The microbiota of eight species of dehydrated edible seaweeds from North West Spain. Food Microbiol..

[42] Ozturkoglu-Budak S., Wiebenga A., Bron P.A., de Vries R.P. (2016). Protease and lipase activities of fungal and bacterial strains derived from an artisanal raw ewe’s milk cheese. Int. J. Food Microbiol..

[43] Antunes-Rohling A., Calero S., Halaihel N., Marquina P., Raso J., Calanche J., Beltrán J.A., Álvarez I., Cebrián G. (2019). Characterization of the spoilage microbiota of Hake Fillets packaged under a modified atmosphere (MAP) rich in CO_2_ (50% CO_2_/50% N_2_) and stored at different temperatures. Foods.

[44] Broekaert K., Noseda B., Heyndrickx M., Vlaemynck G., Devlieghere F. (2013). Volatile compounds associated with *Psychrobacter* spp. and *Pseudoalteromonas* spp., the dominant microbiota of brown shrimp (*Crangon crangon*) during aerobic storage. Int. J. Food Microbiol..

[45] Albakosh M.A., Naidoo R.K., Kirby B., Bauer R. (2015). Identification of epiphytic bacterial communities associated with the brown alga *Splachnidium rugosum*. Environ. Boil. Fishes.

[46] Arahal D.R., Castillo A.M., Ludwig W., Schleifer K.H., Ventosa A. (2002). Proposal of Cobetia marina gen. nov., comb. nov., within the family *Halomonadaceae*, to include the species *Halomonas marina*. Syst. Appl. Microbiol..

[47] Martin M., Barbeyron T., Martin R., Portetelle D., Michel G., Vandenbol M. (2015). The cultivable surface microbiota of the brown alga *Ascophyllum nodosum* is enriched in macroalgal-polysaccharide-degrading bacteria. Front. Microbiol..

[48] Mulaosmanovic E., Lindblom T., Windstam S., Bengtsson M., Rosberg A., Mogren L., Alsanius B. (2021). Processing of leafy vegetables matters: Damage and microbial community structure from field to bag. Food Control.

[49] Tatsika S., Karamanoli K., Karayanni H., Genitsaris S. (2019). Metagenomic characterization of bacterial communities on ready-to-eat vegetables and effects of household washing on their diversity and composition. Pathogens.

[50] Söderqvist K., Osman O.A., Wolff C., Bertilsson S., Vågsholm I., Boqvist S. (2017). Emerging microbiota during cold storage and temperature abuse of ready-to-eat salad. Infect. Ecol. Epidemiol..

[51] Del Olmo A., Picon A., Nuñez M. (2019). High pressure processing for the extension of *Laminaria ochroleuca* (kombu) shelf-life: A comparative study with seaweed salting and freezing. Innov. Food Sci. Emerg. Technol..

[52] Odeyemi O.A., Alegbeleye O.O., Strateva M., Stratev D. (2020). Understanding spoilage microbial community and spoilage mechanisms in foods of animal origin. Compr. Rev. Food Sci. Food Saf..

[53] Fu K., Li J., Wang Y., Liu J., Yan H., Shi L., Zhou L. (2016). An innovative method for rapid identification and detection of vibrio alginolyticus in different infection models. Front. Microbiol..

[54] Dong Y., Zhao P., Chen L., Wu H., Si X., Shen X., Shen H., Qiao Y., Zhu S., Chen Q. (2020). Fast, simple and highly specific molecular detection of *Vibrio alginolyticus* pathogenic strains using a visualized isothermal amplification method. BMC Veter. Res..

[55] Harrysson H., Krook J.L., Larsson K., Tullberg C., Oerbekke A., Toth G., Pavia H., Undeland I. (2021). Effect of storage conditions on lipid oxidation, nutrient loss and colour of dried seaweeds, *Porphyra umbilicalis* and *Ulva fenestrata*, subjected to different pretreatments. Algal Res..

[56] Santiago A., Moreira R., Torres M.D., Kraan S., Dominguez H. (2020). Drying of edible seaweeds. Sustainable Seaweed Technologies.

[57] Badmus U.O., Taggart M., Boyd K.G. (2019). The effect of different drying methods on certain nutritionally important chemical constituents in edible brown seaweeds. Environ. Boil. Fishes.

[58] Tello-Ireland C., Lemus-Mondaca R., Vega-Galvez A., López J., Di Scala K. (2011). Influence of hot-air temperature on drying kinetics, functional properties, colour, phycobiliproteins, antioxidant capacity, texture and agar yield of alga *Gracilaria chilensis*. LWT.

[59] Gupta S., Rajauria G., Abu-Ghannam N. (2010). Study of the microbial diversity and antimicrobial properties of Irish edible brown seaweeds. Int. J. Food Sci. Technol..

[60] Cox S., Gupta S., Abu-Ghannam N. (2012). Effect of different rehydration temperatures on the moisture, content of phenolic compounds, antioxidant capacity and textural properties of edible Irish brown seaweed. LWT.

