# Supplementary material for: Quality and Safety Assessment of Edible Seaweeds Alaria esculenta and Saccharina latissima Cultivated in Scotland
Source: Foods. 2021 Sep 17;10(9):2210. doi: 10.3390/foods10092210 (PMC8472205; doi:10.3390/foods10092210)
Supplement: Supplementary file 1 [file foods-10-02210-s001.zip › foods-1364002-supplementary.pdf]

Supplementary data:

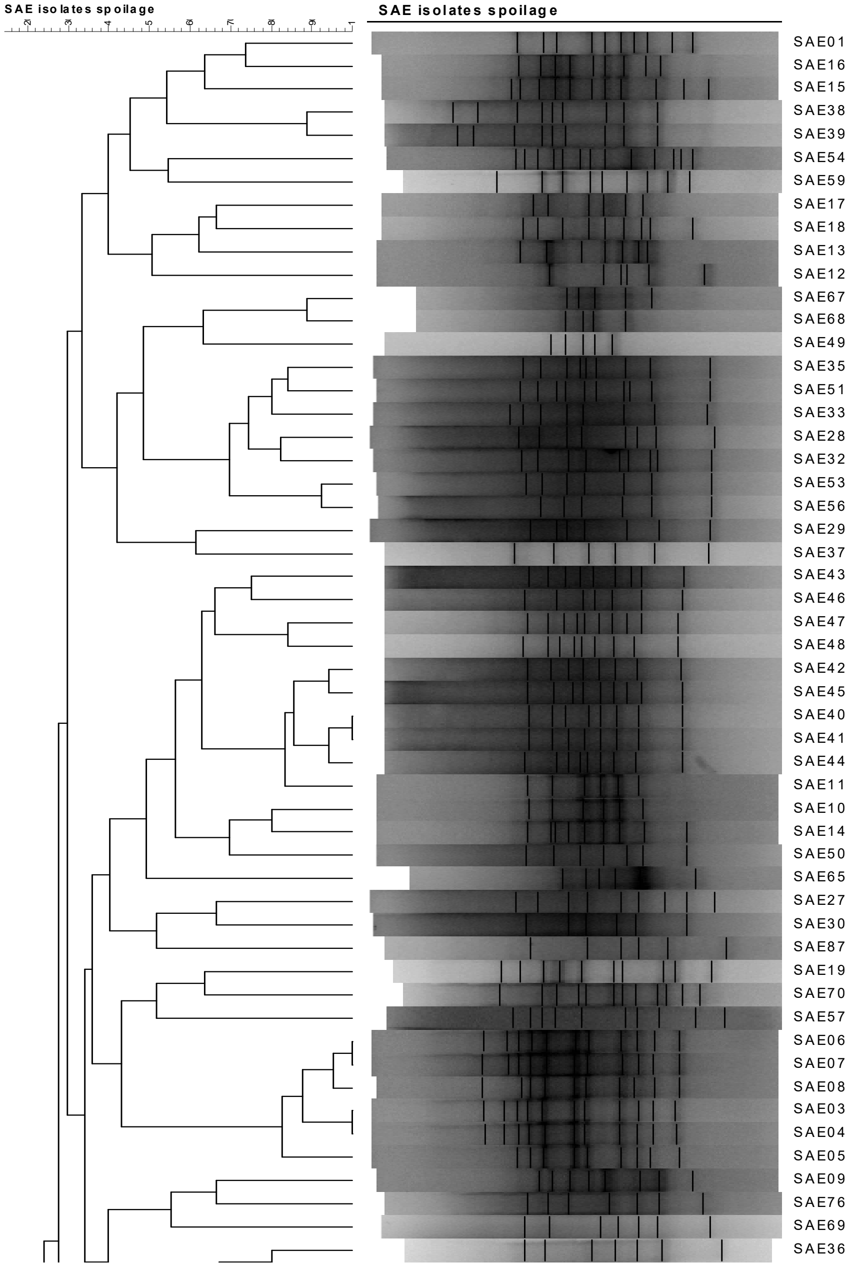

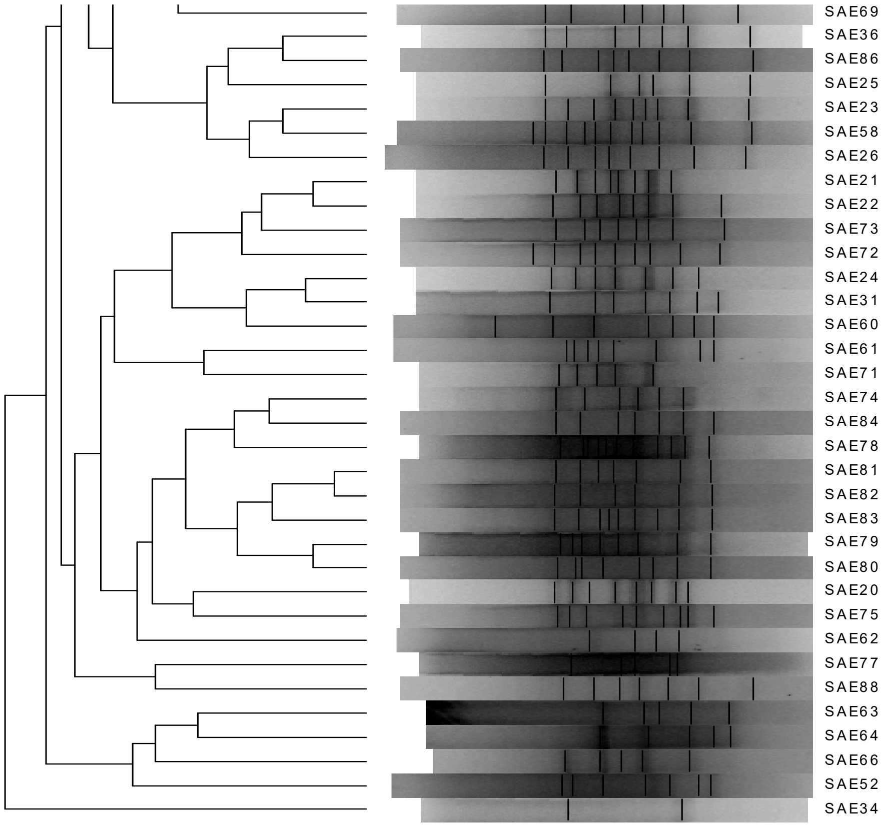

Figure S1. RAPD-PCR clustering of bacteria isolated from *A. esculenta* (fingerprints analyzed with Dice coefficient and dendrogram constructed with UPGMA)

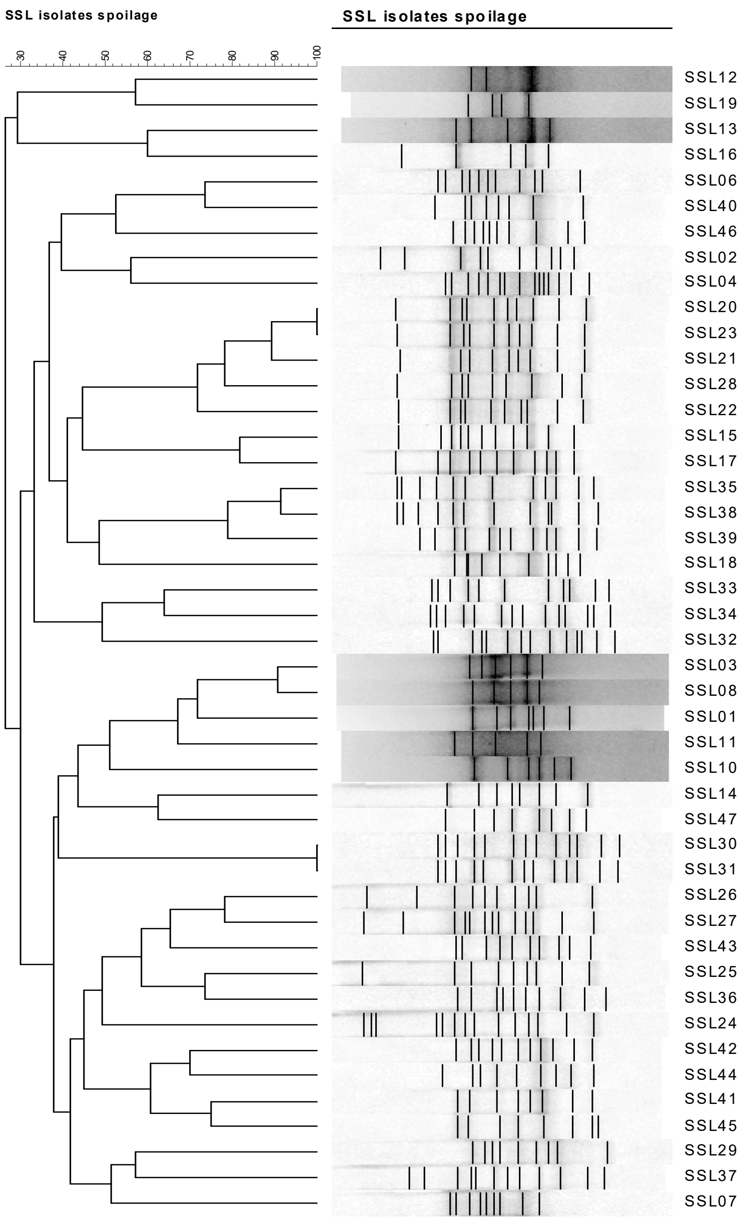

Figure S2. RAPD-PCR clustering of bacteria isolated from *S. latissima* (fingerprints analyzed with Dice coefficient and dendrogram constructed with UPGMA)
